# Giraffe: A tool for comprehensive processing and visualization of multiple long-read sequencing data

**DOI:** 10.1016/j.csbj.2024.08.003

**Published:** 2024-08-09

**Authors:** Xudong Liu, Yanwen Shao, Zhihao Guo, Ying Ni, Xuan Sun, Anskar Yu Hung Leung, Runsheng Li

**Affiliations:** a Department of Infectious Diseases and Public Health, Jockey Club College of Veterinary Medicine and Life Sciences, City University of Hong Kong, Hong Kong, China; b Department of Biomedical Sciences, Jockey Club College of Veterinary Medicine and Life Sciences, City University of Hong Kong, Hong Kong, China; c Department of Medicine, School of Clinical Medicine, Li Ka Shing Faculty of Medicine, The University of Hong Kong, Pok Fu Lam, Hong Kong Special Administrative Region; d ZeBlast Technology Limited, Hong Kong Science Park, Hong Kong Special Administrative Region; e Centre for Oncology and Immunology, Hong Kong Science Park, Hong Kong Special Administrative Region; f Department of Precision Diagnostic and Therapeutic Technology, City University of Hong Kong Shenzhen Futian Research Institute, Shenzhen, China; g Tung Biomedical Sciences Centre, City University of Hong Kong, Hong Kong, China

**Keywords:** Long read sequencing, Direct RNA sequencing, Read quality comparison, Sequencing bias, DNA methylation

## Abstract

Third-generation sequencing techniques have become increasingly popular due to their capacity to produce long, high-quality reads. Effective comparative analysis across various samples and sequencing platforms is essential for understanding biological mechanisms and establishing benchmark baselines. However, existing tools for long-read sequencing predominantly focus on quality control (QC) and processing for individual samples, complicating the comparison of multiple datasets. The lack of comprehensive tools for data comparison and visualization presents challenges for researchers with limited bioinformatics experience. To address this gap, we present Giraffe (https://github.com/lrslab/Giraffe_View), a Python3-based command-line tool designed for comparative analysis and visualization across diverse samples and platforms. Giraffe facilitates the assessment of read quality, sequencing bias, and genomic regional methylation proportions for both DNA and direct RNA sequencing reads. Its effectiveness has been demonstrated in various scenarios, including comparisons of sequencing methods (whole genome amplification vs. shotgun), sequencing platforms (Oxford Nanopore Technology, ONT vs. Pacific Biosciences, PacBio), tissues (kidney marrow with and without blood), and biological replicates (kidney marrows).

## 1 Introduction

Comparative analysis of samples collected from different individuals or generated from different sequencing platforms is crucial for exploring biological mechanisms and establishing benchmark baselines. Third-generation sequencing platforms, such as Oxford Nanopore Technologies (ONT) and Pacific Biosciences (PacBio), have gained significant popularity in biological research due to their ability to generate long and high-quality reads [1], [2], [3], [4], [5], [6]. However, current tools for quality control and long-read assessment, such as pycoQC [7] and minion_qc [8], are primarily designed to analyze in-house sequencing data and handle individual samples, lacking the capability for comparative analysis across multiple samples and platforms.

To address this gap, we developed Giraffe, a set of Python3-based scripts specifically designed for multiple long-read data comparisons in read quality, sequencing bias, and distribution of genomic regional methylation proportion. Compared to alternative tools like NanoComp, an effective package that provides essential metrics such as read length and Q values [9], Giraffe provides greater features and options. These features include estimated and observed read accuracy as well as homopolymer identification. All these metrics help users understand their data quality and determine whether the quality is reliable enough for downstream analysis.

## 2 Materials and methods

### 2.1 Pre-processing example data

The raw current signal files in fast5 format generated from the ONT platform were profiled to get the base and methylation information using Dorado V0.5.3 (https://github.com/nanoporetech/dorado).

For the read basecalling model, dna_r10.4.1_e8.2_400bps_sup@v4.3.0 and dna_r9.4.1_e8_sup@v3.6 were used for R10.4.1 and R9.4.1 data, respectively. For the methylation basecalling model, dna_r9.4.1_e8_sup@v3.3_5mCG@v0.1 and dna_r10.4.1_e8.2_400bps_sup@v4.3.0_5mC_5hmC@v1 were used for R9.4.1 and R10.4.1 data, respectively.

### 2.2 Computing estimating read accuracy

To estimate the read accuracy, we first converted the Q score associated with each base into an error probability, thereby quantifying the likelihood of an error occurring at that specific base position within a read. Then, the average error probability across all bases within a given read was computed and served as a comprehensive representation of the read error probability. To derive the estimated read accuracy, we subtracted the read error probability from 1, as indicated by Eq. 1.

(1) $$\mathrm{Estimated read accuracy}=1-\left[\frac{1}{N}*\sum 10^{{-q}_{i}/10}\right]$$

Here, N represents the total number of bases within a read, and $q_{i}$ denotes the base quality score (Phred score or Q value) assigned to the *i*-th basecall within the read. This equation was well applied to zebrafish and human samples [5], [6].

### 2.3 Computing observed read quality and mismatch proportion

To get the observed read quality such as accuracy and mismatches proportion, the reads were aligned against their reference genome using Minimap2 (V2.17-r941) [10], [11]. The different alignment settings were used for different sequencing platforms, the "map-ont" and "map-pb" were used for ONT and PacBio sequencing platforms, respectively. As for the ONT direct RNA sequencing reads, the "-ax splice -uf -k14" was used. The resulting alignment SAM file was sorted and converted into the BAM file using samtools (V1.17) [12]. The Python package pysam [12], [13], [14], [15] was utilized to load the BAM file and take the alignment features. Next, we summarized the length of substitution, insertion, deletion, and matched base within the read and computed the observed accuracy (Eq. 3) and mismatches proportion ((5), (6), (7)).

(2) $$N\left(\mathrm{total}\right)=N\left(\mathrm{sub}\right)+N\left(\mathrm{mat}\right)+N\left(\mathrm{ins}\right)+N\left(\mathrm{del}\right)$$

(3) $$\mathrm{Observed read accuracy}=N\left(\mathrm{mat}\right)/N\left(\mathrm{total}\right)$$

(4) $$\mathrm{Observed identification}=N(\mathrm{mat})/\left[N\left(\mathrm{mat}\right)+N(\mathrm{sub})\right]$$

(5) $$\mathrm{Insertion proportion}=N\left(\mathrm{ins}\right)/N\left(\mathrm{total}\right)$$

(6) $$\mathrm{Deletion proportion}=N\left(\mathrm{del}\right)/N\left(\mathrm{total}\right)$$

(7) $$\mathrm{Substitution proportion}=N\left(\mathrm{sub}\right)/N\left(\mathrm{total}\right)$$

Here $N\left(\mathrm{mat}\right)$, $N\left(\mathrm{ins}\right)$, $N\left(\mathrm{del}\right)$, and $N\left(\mathrm{sub}\right)$ were length of matched base, insertion, deletion, and substitution within the read. Those equations were well applied to zebrafish and human samples [5], [6].

### 2.4 Calculating sequencing bias

To detect the sequencing bias within sequencing data, we divided the genome into multiple bins first. The bin size was set to a default value of 1 kb, the user could also provide a different bin size if desired. Within these bins, the “gcbias” function could calculate the GC content and sequencing depth. The bins were then categorized based on their GC content range from 0 to 100 %. Then we summarized the distribution of these bins and determined the average read coverage for each GC content range. To ensure effective analysis, the function selected a scale that encompasses over 90 % of the bins, as most of the GC content falls within smaller ranges rather than spanning the entire 0 to 100 % spectrum. Sequencing depth was subsequently normalized within this selected scale.

### 2.5 Profiling the methylation information

After modification calling, the generated modBAM files with methylation information were subsequently processed using modkit (V0.3.0) (https://github.com/nanoporetech/modkit) with the parameters of “pileup” and “--cpg”. The 18 column bedMethyl files would be generated with the format suggested by ONT (https://nanoporetech.github.io/modkit/intro_bedmethyl.html). For the R9.4.1 datasets, all profiled methylation sites were identified as 5-methylcytosine (5mC) within CpG contexts. In contrast, for the R10.4.1 datasets, both 5-hydroxymethylcytosine (5hmC) and 5mC were profiled. In the generated bedMethyl files, 5hmC sites were denoted by the signal “h”, while 5mC sites were indicated by “m”. Both 5mC and 5hmC methylation files are supported as input to the “modbin” function. Here, we used the 5mC proportion at targeted regional levels as examples. Based on these different signals, the 5mC sites were distinguished. For the subsequent analysis, the bedMethyl files were filtered to retain only positions including chromosomes, start, and end, along with the methylation proportion. These filtered data were then used as input for the “modbin” function in Giraffe.

## 3 Results and discussion

### 3.1 Software description

**Fig. 1:**
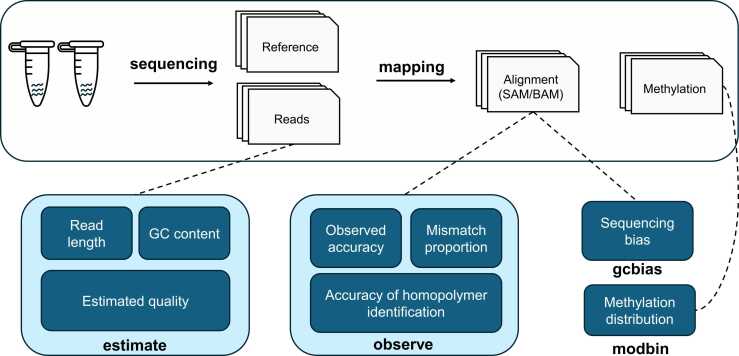
The workflow of the Giraffe. The basic information included in length, GC content, and estimated accuracy for each read can be profiled by the “estimate” function. After aligning reads against the reference, observed accuracy, mismatch proportion, and accuracy of homopolymer identification are calculated by the “observe” function. The alignment files can be also used by the “gcbias” function to detect the sequencing bias. As for the genomic regional methylation proportion, it can be summarized by the “modbin” function.

Giraffe has four functions, including “estimate” for estimated read quality assessment, “observe” for observed read accuracy evaluation, “gcbias” for sequencing bias detection, and “modbin” for genomic regional methylation proportion comparison (Fig. 1). Giraffe supports multi-dataset comparisons, with theoretically no limit on the number of datasets that can be analysed. The user has two strategies for applying Giraffe to their datasets. The first strategy is the one-button pattern, where Giraffe supports input in the form of FASTQ reads or unaligned SAM/BAM files and conducts three functions related to read quality assessment: "estimate", "observe", and "gcbias". Upon completion of the analysis, Giraffe generates an HTML summary that presents the statistical results and related figures. The second strategy is that the user can run the four functions independently according to their specific needs. The demo data for users testing Giraffe are provided with the subfunction “giraffe_run_demo”, which can automatically download the demo and run the four core functions independently and the one-button pattern. The demo datasets include ONT R10.4.1 (79 MB) and R9.4.1 (121 MB) reads collected from *Escherichia coli* with a reference (4.8 MB) and 5mC methylation files profiled from zebrafish blood (23 MB) and kidney (19 MB) samples. The detailed results are available in the directory named “Giraffe_Results” (Tables S1-S7). A demonstration of the HTML summary is available at https://lxd98.github.io/giraffe.github.io.

### 3.2 Installation and dependencies

Giraffe and the associated Python scripts are conveniently accessible through the public software repositories conda (https://conda.org) and PyPI (https://pypi.org). To support the read processing functionalities, Giraffe relies on dependencies including minimap2 (V2.17-r941) [10], [11], samtools (V1.17) [15], and bedtools (V2.30.0) [16], which can be obtained through conda. The scripts are developed utilizing several Python modules, including pysam [12], [13], [14], [15], seaborn (https://github.com/mwaskom/seaborn), numpy [17], pandas (https://github.com/pandas-dev/pandas), Biopython (https://biopython.org), and termcolor (https://github.com/termcolor/termcolor). Users can install Giraffe and its dependencies directly using conda, or alternatively, users can independently install the dependencies and then install Giraffe via pip in Python.

### 3.3 Estimated read quality

The “estimate” function provides the user with basic information, generating a table that includes estimated accuracy, estimated error proportion, Q score, length, and GC content for each read (Table S1). The estimated read accuracy, a value ranging from 0 to 100 %, is transformed from the Q score (Phred score) to facilitate simpler and more intuitive comparisons of data quality (Eq. 1). The function requires input from a table containing sample ID, sequencing platform (ONT/ONT_RNA/Pacbio), and the path of the data in FASTQ or unaligned SAM/BAM format. Also, the FASTQ files can be compressed via gzip or not. When the --plot parameter is specified, the estimate function will generate three figures: a density plot of read accuracy, a density plot of read length, and a boxplot of read GC content.

### 3.4 Observe read accuracy

The Q score is calculated by the estimated probability of the base being wrong, which is commonly used to quantify the quality of baseballs in sequencing data. However, there are situations, such as overfitting of the basecalling model, where the Q score may not accurately reflect the read quality [6], which may inflect the parameter options for the genome assembler like “--nano-raw” for ONT read with less 20 % estimated error and “--nano-hq” for ONT read with less 5 % error in Flye [18]. Thus, the “observe” function based on the read alignment is developed ((2), (3), (4), (5), (6), (7)**)**. Considering that detecting the homopolymer in long-read sequencing accurately is still challenging [19], homopolymer identification is also included in the “observe” function as a comparative metric. The “observe” supports three types of input including FASTQ reads, unaligned SAM/BAM files, and aligned SAM/BAM files. For the former two input types, a reference is also needed. After finishing the analysis, two summary tables will be generated, one specifically for homopolymer identification and another for detailed read accuracy such as length of insertions, deletions, substitutions, and matches, as well as the observed read accuracy (Tables S2 and S3). The results can be visualized by specifying the parameter of “--plot”.

In observed read accuracy calculations, a potential issue is that real single nucleotide polymorphisms (SNPs) or indels differing from the reference will also be included. The true variations will be counted as read errors, which might have an impact on the result of the Giraffe. The over-estimation of read error for distal individuals from reference genome is a common issue for all quality control pipelines. In our results, we only showed the comparison using the same DNA source, which was from the same cell line or individuals. The heterozygosity levels were expected to be quite low in these DNA samples. For DNA samples with high heterozygosity, we would recommend using a high-quality pan-genome reference that includes multi-haplotype information to carry out read alignment. For a more precise read accuracy estimation, users can also mask regions with SNPs or indels after read alignment and input the filtered BAM files into Giraffe for further analysis.

### 3.5 Sequencing bias

In wet lab experiments, some processes can introduce sequencing bias such as polymerase chain reaction (PCR). More specifically, during DNA amplification, the use of non-random primers or different affinities of polymerase enzyme to DNA sequences can result in uneven coverage and increased depth of specific regions. The results generated by analyzing data with a sequencing bias are unreliable, especially for methods based on counting. The “gcbias” function was designed to assess the relationship between GC content and sequencing depth, reflecting the level of sequencing bias. The function requires the sorted SAM/BAM files to calculate the sequencing coverage and reference to calculate the GC content.

### 3.6 Genomic regional methylation profiling

The methylation proportion in eukaryotes is associated with the strength of promoter activity and downstream protein expression, which has significant implications for exploring the mechanisms underlying various diseases [20], [21]. Nanopore sequencing for native DNA can now give accurate 5mC methylation profiling for every CpG site, especially for human samples. The methylation level for each functional region would be required for downstream analysis. The “modbin” function was designed to facilitate the comparison of methylation at the genomic regional level and detect the difference between samples. The modbin function requires files containing site-level methylation data and a table with the target regions. Users need to profile the methylation data from the modBAM files independently. We provide a protocol to achieve this in the Giraffe documentation.

### 3.7 Additional scripts and options

Considering that the scales of normalization may not be reasonable or suitable for all users in the sequencing bias detection part, additional scripts, including “renormalization_sequencing_bias” and “giraffe_plot”, are provided to allow users to manually define the normalized scale based on the distribution of bins and replot figure. Additionally, another script, named “homopolymer_count”, is offered to count the number, position, and type of homopolymers in the reference genome.

### 3.8 Application of Giraffe to different samples

Here, we showcased four applications of Giraffe: exploring the influence of amplification, benchmarking the read quality between different sequencing platforms, identifying the influence of sample purity on sequencing data, and investigating the consistency of biological replicates. The details of all datasets are available in Table S8.

**Fig. 2:**
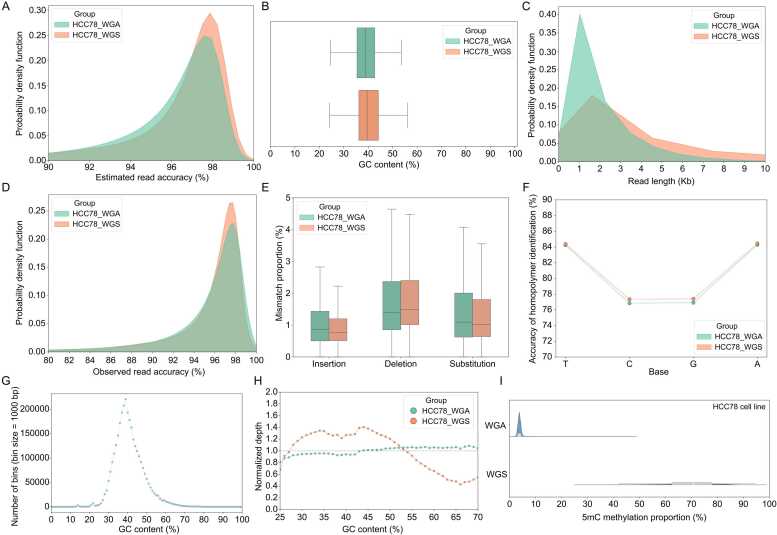
Comparison of human HCC78 cell line between WGS and WGA reads. (**A**), (**B**), and (**C**) the distribution of estimated accuracy, GC content, and length. (**D**) and (**E**) the distribution of observed accuracy and mismatch proportion. (**F**) The accuracy of homopolymer identification for each base type. (**G**) The distribution of 1k bin numbers within 0 to 100 % GC content. (**H**) Relationship between sequencing depth and GC content at 1k bin level. (**I**) The distribution of 5mC methylation proportion at 1 M bin level. WGS: whole genome sequencing; WGA: whole-genome amplification.

DNA amplification is an essential step before obtaining genome information from limited sample availability. To evaluate the impact of amplification on sequencing reads, DNA samples with and without whole genome amplification derived from the human HCC78 cell line were sequenced using ONT platforms with R9.4.1 flow cells. Upon observation, it became apparent that the amplification process had minimal impact on the sequencing quality and GC content (Figs. 2**A**, 2**B**, and 2**D**-**2F**). However, a noticeable bias was identified in the amplified data (Fig. 2**H**). In terms of methylation proportion (5mC) at the 1 M bin level, a significant majority of the bins derived from amplified reads clustered near 0 % methylation (Fig. 2**I**).

Different long-read sequencing platforms offer distinct advantages depending on the specific requirements. To assess the read accuracy of long-read sequencing platforms, the ONT duplex reads and PacBio HiFi reads derived from human HG002 datasets were employed. Remarkably, both datasets achieved exceptional accuracy levels exceeding 99 % and showcased similar GC content of approximately 40 % (Fig. S1A, S1B, and S1D). Notably, the PacBio reads exhibited an enrichment of around 20 Kb in read length, whereas the ONT reads displayed a more evenly distributed range (Fig. S1C). Furthermore, in comparison to HiFi reads, duplex reads demonstrated superior performance in homopolymer identification and less bias in terms of read coverage across the reference genome (Fig. S1F and S1H).

Different tissues within a biological organism generally exhibit distinct patterns of protein expression and methylation proportion due to their specialized functions. However, isolating a specific tissue without any contamination from other tissues can be challenging in practice. To investigate the impact of other tissue contamination on downstream analysis results, the ONT R9.4.1 reads obtained from the kidney marrow of zebrafish, with and without the presence of blood, were employed. A clear observation was made regarding the decreased quality of kidney marrow with blood (KMB) reads (Fig. S2A and S2D). The decrease in quality was evidenced by higher mismatch proportion and low homopolymer identification accuracy (Fig. S2E and S2F).

Biological systems are complex and can be influenced by numerous variables, making it difficult to isolate and control a single changing factor in experiments. The biological replicates are necessary to evaluate the consistency and reproducibility of their results. Here, two sets of zebrafish kidney marrow duplicate samples sequenced by ONT platforms equipped with R10.4.1 flow cells were used for evaluation. The similar read quality, GC content, and methylation pattern of the two samples can be regarded as important metrics of being a qualified replication (Fig. S3).

### 3.9 Resource usage of Giraffe to various data sizes

To establish a baseline for resource usage during the execution of Giraffe with varying data sizes, HG002 human duplex reads were subsampled into chunks of 0.5, 1, 2, 5, 10, 20, and 50 GB in FASTQ format (Table S9). Performance was assessed using two metrics: runtime and maximum physical memory usage. All functions were executed with default parameters and 24 processes (see Supplementary Material). The testing was conducted on a workstation equipped with two Intel® Xeon® Platinum 8160 processors (48 cores, 96 threads) and 345 GB of memory (2133 MHz).

The "estimate" function required approximately 8 min and a maximum of 2.6 GB of memory to process the 50 GB FASTQ dataset (Table S10 and S11). For the "observe" function, two input strategies were compared: direct FASTQ reads input and sorted BAM file input, both derived from the same FASTQ files. The FASTQ input took about 1 h and 55 min, roughly 2.3 times longer than the BAM input (48 min), with a maximum physical memory usage (22.43 GB) being 1.5 times higher than the BAM input (14.45 GB) for the 50 GB datasets (Table S10 and S11). The mapping and processing of BAM files, including merging and sorting, demanded substantial time and memory. In Giraffe, mapping was conducted using minimap2 without specific controls for memory usage. For users with limited memory resources, particularly for large datasets, it is recommended to run the mapping task independently using the "--split-prefix=tmp" parameter in minimap2, which reduces memory usage but is slower and requires temporary disk space. Additionally, users should include the "--MD" and "--secondary=no" parameters in independent mapping, with the former being crucial for counting read substitutions and the latter for suppressing secondary alignments. The sorted BAM files can then be directly used for subsequent Giraffe analyses. The "gcbias" function exhibited a consistent maximum physical memory usage (1.66 GB) across all dataset sizes, correlating with the number of processes used (Table S10 and S11). The "gcbias" required approximately 48 min to analyze the 50 GB dataset. Since "modbin" processes site methylation information rather than FASTQ or SAM/BAM files, this function was tested separately (Table S10 and S11). It required about 77 s and a maximum physical memory of 2.88 GB to handle the 1.5 GB methylation file at the 1 M bin level (3132 bins) (Table S10 and S11).

## 4 Conclusion

Giraffe is a tool designed for comparing multiple long-read sequencing samples. It offers four main functions: “estimate”, “observe”, “gcbias”, and “modbin” involving read quality, sequencing bias, and genomic regional methylation proportion. Additionally, the usability and applicability of the Giraffe are well-validated in different research backgrounds.

## Funding

This work was supported by the Early Career Scheme from the Research Grants Council of the Hong Kong Special Administrative Region, China (Project No. CityU 21100521); the Hong Kong Health and Medical Research Fund (project number 08194126); Hetao Shenzhen-Hong Kong Science and Technology Innovation Cooperation Zone Shenzhen Park Project (HZQB-KCZYZ-2021017); new Research Initiatives support from 10.13039/100007567City University of Hong Kong (project number 9610497) to R.L; and was supported by the Theme-based Research Scheme (T12-702/20-N), Health and Medical Research Fund Projects No.08192066 and No. 08193106, 10.13039/501100001809National Natural Science Foundation of China (NSFC)/Research Grants Council (RGC) Joint Research Scheme 2021/22 N_HKU745/21, and the National Key Research and Developmen Program of China (2023YFA1800100) to A.L.

## CRediT authorship contribution statement

**Xuan Sun:** Writing – review & editing, Data curation. **Ying Ni:** Writing – review & editing, Data curation. **Runsheng Li:** Writing – review & editing, Validation, Supervision, Methodology, Investigation, Funding acquisition, Conceptualization. **Anskar Yu Hung Leung:** Writing – review & editing, Supervision, Funding acquisition. **Xudong Liu:** Writing – review & editing, Writing – original draft, Validation, Software, Methodology, Investigation, Formal analysis, Data curation, Conceptualization. **Zhihao Guo:** Writing – review & editing, Methodology. **Yanwen Shao:** Writing – review & editing, Validation, Investigation, Data curation.

## Declaration of Competing Interest

The authors declare that they have no known competing financial interests or personal relationships that could have appeared to influence the work reported in this paper.

## Data Availability

The source code is available at https://github.com/lrslab/Giraffe_View and PyPI (https://pypi.org/project/Giraffe-View). All the datasets used in this work can be found in Table S8. The PacBio HiFi and ONT duplex reads are published, which are available at https://humanpangenome.org/data. The other three datasets can be found at the National Centre for Biotechnology Information (NCBI) with project ID PRJNA1095401. The testing demo datasets are available in Figshare (https://figshare.com/articles/dataset/demo_datasets/25378693).
