# Supplementary material for: Giraffe: A tool for comprehensive processing and visualization of multiple long-read sequencing data
Source: Comput Struct Biotechnol J. 2024 Aug 9;23:3241–6. doi: 10.1016/j.csbj.2024.08.003 (PMC11393587; doi:10.1016/j.csbj.2024.08.003)
Supplement: Supplementary file 1 — Supplementary material. [file mmc1.docx]

**Supplementary Materials**

The commands for function testing on datasets of varying sizes. The metrics includes the time usage and maximum physical memory usage.

# Estimate

for i in `ls read_*txt`;do

echo $i

/usr/bin/time -v giraffe estimate --read $i --cpu 24

done

# Observe (FASTQ input)

for i in `ls read_*txt`;do

echo $i

/usr/bin/time -v giraffe observe --read $i --ref GCF_009914755.1_genomic.fa --cpu 24

done

# Observe (aligned BAM input)

for i in `ls aligned_*txt`;do

echo $i

/usr/bin/time -v giraffe observe --aligned $i --cpu 24

done

# GC bias

for i in `ls aligned_*txt`;do

echo $i

/usr/bin/time -v giraffe gcbias --aligned $i --ref GCF_009914755.1_genomic.fa --cpu 24

done

**Supplementary Figures**

**
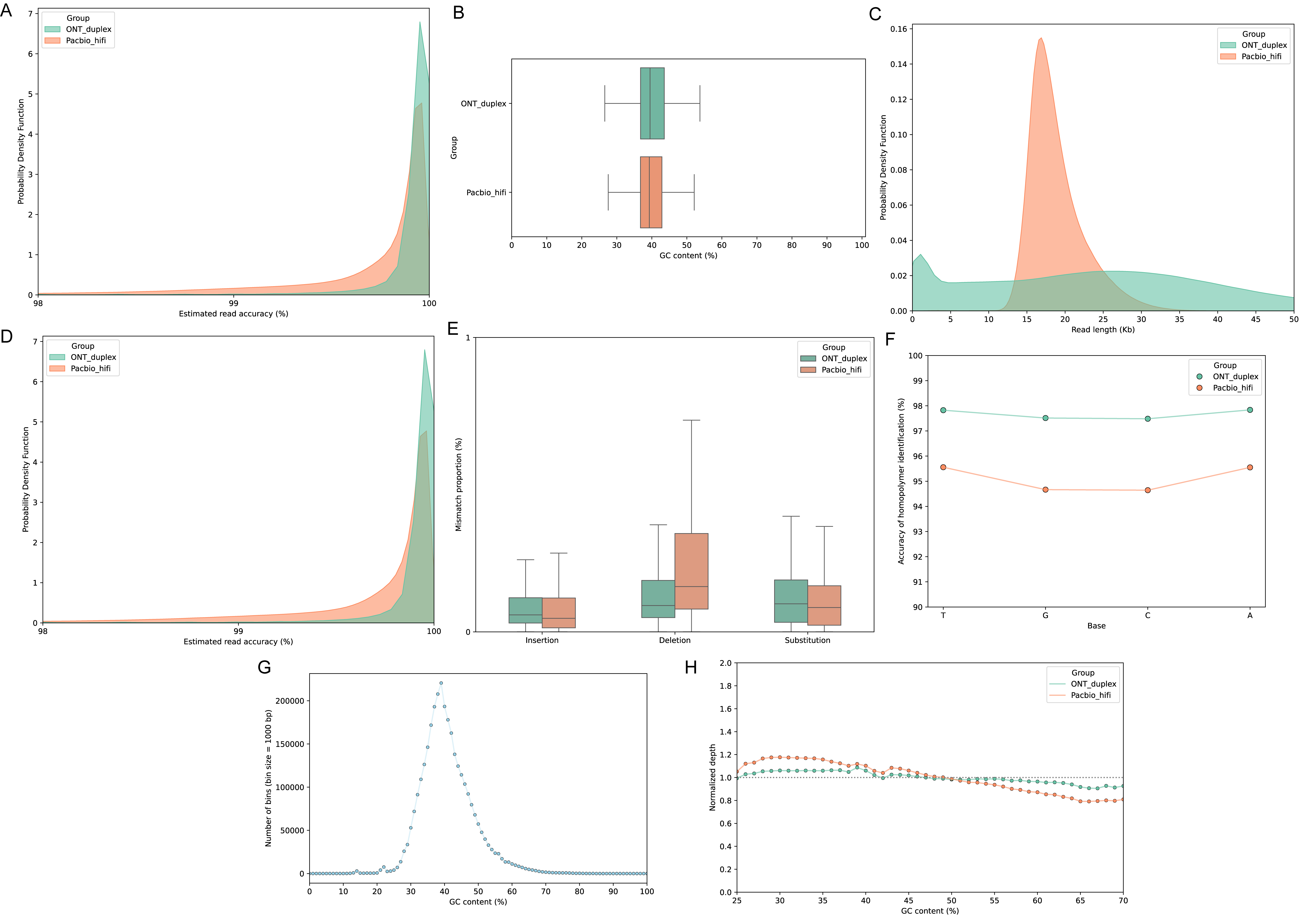
**

**Figure S1.** Comparison of human HG002 PacBio HiFi and ONT duplex data. (**A**), (**B**), and (**C**) the distribution of estimated accuracy, GC content, and length for each read, respectively. (**D**) and (**E**) the distribution of observed accuracy and mismatch proportion. (**F**) The accuracy of homopolymer identification for each base type. (**G**) The distribution of 1kp bin numbers within 0 to 100% GC content. (**H**) Relationship between sequencing depth and GC content at 1k bin level.


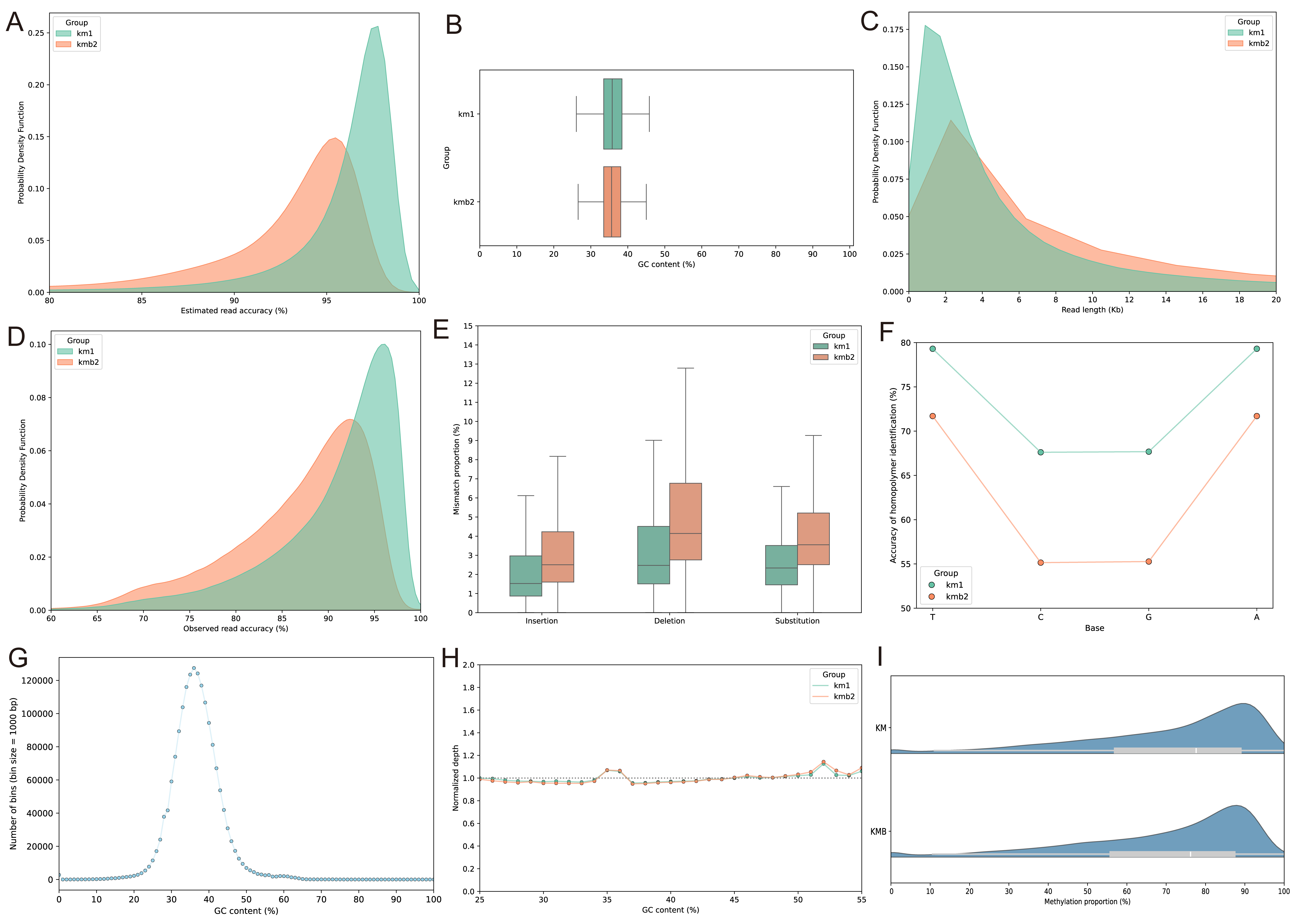


**Figure S2.** Comparison of zebrafish kidney marrow data, with or without blood. (**A**), (**B**), and (**C**) the distribution of estimated accuracy, GC content, and length for each read, respectively. (**D**) and (**E**) the distribution of observed accuracy and mismatch proportion. (**F**) The accuracy of homopolymer identification for each base type. (**G**) The distribution of 1kp bin numbers within 0 to 100% GC content. (**H**) Relationship between sequencing depth and GC content at 1k bin level. (**I**) The distribution of methylation proportion at the promoter level. **km:** kidney marrow, **kmb:** kidney marrow with blood.

**
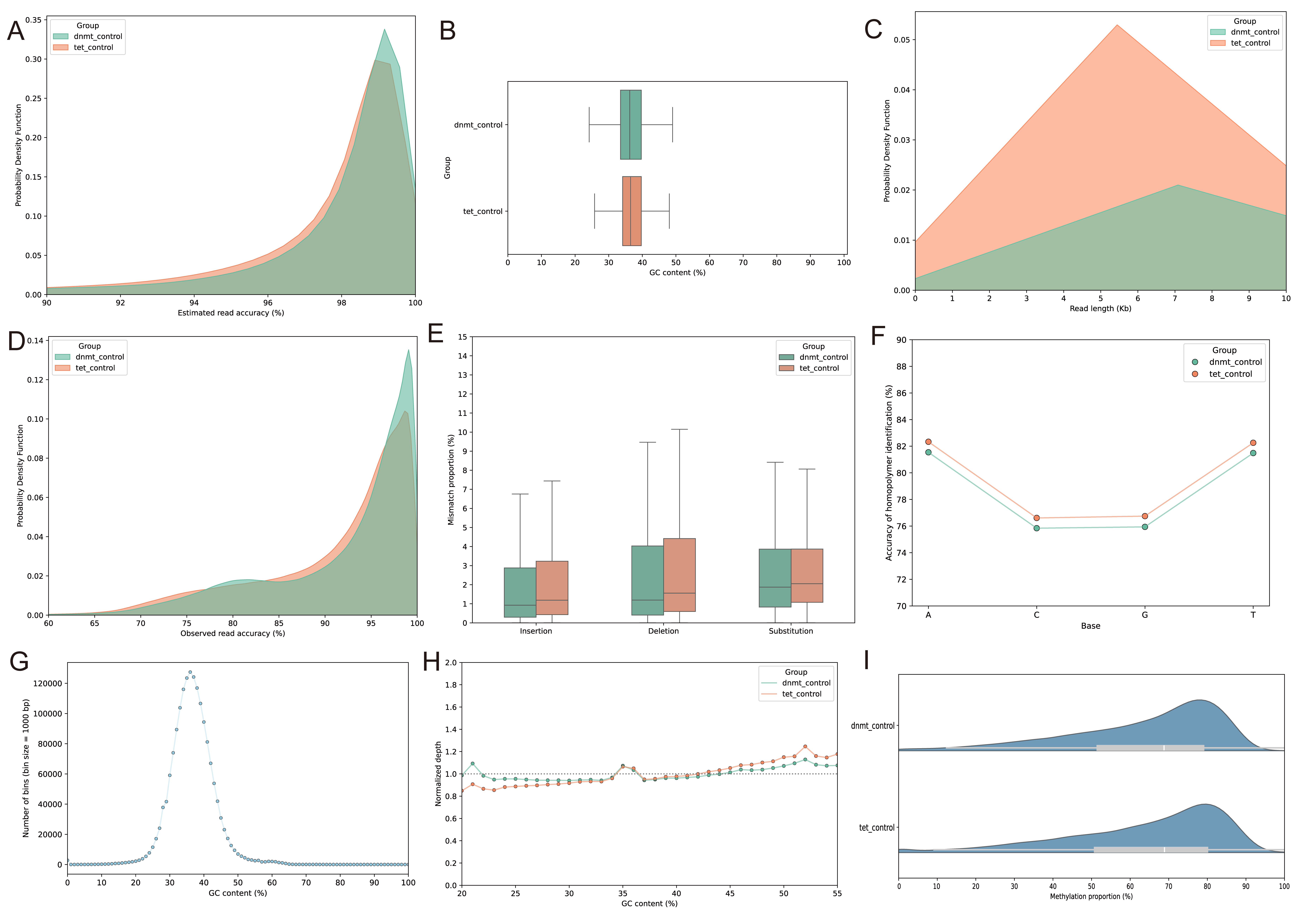
**

**Figure S3.** Comparison of two zebrafish kidney marrow data. Both samples were collected from the wild type. (**A**), (**B**), and (**C**) the distribution of estimated accuracy, GC content, and length for each read, respectively. (**D**) and (**E**) the distribution of observed accuracy and mismatch proportion. (**F**) The accuracy of homopolymer identification for each base type. (**G**) The distribution of 1kp bin numbers within 0 to 100% GC content. (**H**) Relationship between sequencing depth and GC content at 1k bin level. (**I**) The distribution of methylation proportion at the promoter level.
